# New Insights into Pathogenesis and Management of Keratoacanthoma: A Narrative Review

**DOI:** 10.3390/ijms262010040

**Published:** 2025-10-15

**Authors:** Mariafrancesca Hyeraci, Dario Didona, Damiano Abeni, Francesca Magri, Francesco Ricci, Chiara Bertagnin, Arianna Loregian, Giovanni Di Lella, Antonio Di Guardo, Annarita Panebianco, Camilla Chello, Claudio Conforti, Elena Dellambra, Luca Fania

**Affiliations:** 1 Department of Molecular Medicine, University of Padua, 35122 Padua, Italy; chiara.bertagnin@unipd.it (C.B.); arianna.loregian@unipd.it (A.L.); 2 IRCCS Istituto Dermopatico dell’Immacolata (IDI-IRCCS), Dermatological Research Hospital, 00167 Rome, Italy; d.didona@idi.it (D.D.); d.abeni@idi.it (D.A.); f.magri@idi.it (F.M.); f.ricci@idi.it (F.R.); g.dilella@idi.it (G.D.L.); a.diguardo@idi.it (A.D.G.); a.panebianco@idi.it (A.P.); c.chello@idi.it (C.C.); c.conforti@idi.it (C.C.); e.dellambra@idi.it (E.D.); l.fania@idi.it (L.F.); 3 Microbiology and Virology Unit, Padua University Hospital, 35128 Padua, Italy; 4 Department of Life Science, Health and Health Professions, Link University of Rome, 00165 Rome, Italy

**Keywords:** keratoacanthoma, human papillomavirus, non-melanoma skin cancer, dermoscopy, reflectance confocal microscopy, line-field confocal optical coherence tomography, carcinogenesis, therapies, vaccines

## Abstract

Keratoacanthoma (KA) is a rapidly growing epithelial neoplasm characterized by clinical and histopathological features that often overlap with well-differentiated squamous cell carcinoma (SCC), posing diagnostic challenges. This review provides a comprehensive overview of KA, emphasizing advances in non-invasive diagnostic techniques such as dermoscopy, reflectance confocal microscopy (RCM), and line-field confocal optical coherence tomography (LC-OCT), which improve lesion characterization and differentiation from SCC. We discuss the histopathological phases of KA and highlight key features aiding in diagnosis. Furthermore, we explore the emerging role of human papillomavirus (HPV), particularly β-genus types, as a cofactor in KA carcinogenesis through modulation of apoptosis and DNA damage response pathways, especially under ultraviolet (UV) radiation exposure. Therapeutic strategies remain centered on complete surgical excision; however, alternative treatments, including radiotherapy, cryotherapy, topical agents, and systemic retinoids, are discussed with their respective benefits and limitations. Finally, we review current HPV vaccines and novel vaccine candidates targeting a broad spectrum of mucosal and cutaneous HPV types. This review underscores the importance of integrated diagnostic and therapeutic approaches to optimize KA management and highlights future directions in understanding its pathogenesis and treatment.

## 1. Introduction

**Figure 1:**
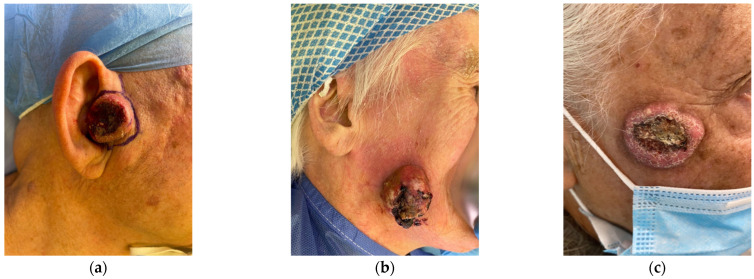
Clinical presentation of three keratoacanthomas. (**a**) Dome-shaped nodule with a central keratin-filled crater located on the right tragus of an elderly man. (**b**) Large exophytic lesion on the right cheek of an elderly man. (**c**) Keratoacanthoma of the right zygomatic-temporal region in an elderly male patient. All three lesions share the classical clinical features of keratoacanthoma: a rapidly growing, skin-colored to erythematous nodule with a characteristic central crater filled with keratin.

Keratoacanthoma (KA) is classified among the non-melanoma skin cancers (NMSC), typically arising on the head and neck areas. It is still controversial whether KA is a distinct neoplasm or a subtype of well-differentiated squamous cell carcinoma (SCC). [1]. Additionally, KA is more common in older adults, particularly those with prolonged sun exposure or immunosuppression, and it occurs more frequently in men than in women [2]. KA is thought to originate from the hair follicle and can appear in solitary, multiple, or eruptive forms, with the solitary type being the most prevalent [2]. Clinically, the most common presentation is a solitary lesion measuring 1 to 2 cm in diameter (Figure 1). Less common variants include giant KA—defined as a solitary tumor larger than 2 cm, typically found on the eyelid or nose—and KA *centrifugum marginatum*, characterized by peripheral expansion with central healing, which can also exceed 2 cm in diameter [3]. Typically, KA progresses through three clinical phases: a proliferative phase, a stabilization phase, and a regression phase [4]. Several signaling pathways have been implicated in KA pathogenesis, including the Wnt/retinoic acid pathway, B-Raf, H-Ras, p27, and the Hedgehog signaling pathway [5,6,7,8].

This article aims to explore the key aspects of KA, including its clinical presentation, pathogenesis, histological aspects, and the available treatment options, providing an insight into the management of this tumor.

## 2. Epidemiology of Keratoacanthoma

Epidemiological data about KA are still inconsistent because of underestimation due to shared morphological features with SCC, especially in the case of well-differentiated SCC. The reported incidence of KA ranges between 100 and 150 cases per 100,000 individuals [9,10]. Recently, the KA peak incidence has shifted toward the age group 65 to 71 years from 50 to 69 years observed in the 1990s [11]. Men are more often affected than women [11]. KA affects mostly fair-skinned people and has not yet been reported in native Australians [9].

## 3. Etiology

The etiology of KA is still not completely understood. It is presumed to originate from the hair follicle, usually located on the hair-bearing, sun-exposed parts of elderly individuals, and it is characterized by a triphasic trend of rapid development, stabilization, and regression, which mimics the hair cycle [12,13]. Multiple factors, also reported for well differentiated SCC [14], have been suggested, including UV radiation exposure, chemical carcinogens, immunosuppression, medications, genetic predisposition such as mutations of p53 or H-Ras, viral infection by HPV, and cutaneous trauma or surgery to the location [15].

In the literature, cases of KA have been reported after COVID-19 vaccination, including both multiple and eruptive KA and solitary KA, strictly located on the site of vaccination [16,17]. It has been hypothesized that the onset of KA after vaccination may have been initiated by an immune-mediated, pro-inflammatory mechanism.

KA is widely reported also after cutaneous trauma, including tattooing [18], site injection of cosmetic collagen filler [19], and at the recipient site of skin graft [20].

KA can also be triggered by medications, including BRAF inhibitors (such as pembrolizumab) [21], but also kinase inhibitor drugs (such as sorafenib), PD-1 inhibitors (such as sintilimab) [22], and immunosuppressive disease-modifying antirheumatic drugs-DMARDs-(such as leflunomide) [23].

KA may also be associated with various genetic syndromes: Muir-Torre syndrome, multiple self-healing squamous epithelioma (MSSE, Ferguson-Smith syndrome), multiple familial KA of Witten and Zak, *xeroderma pigmentosum*, multiple self-healing palmoplantar carcinoma, *incontinentia pigmenti*, and eruptive KA of Grzybowski [24].

Recently, the key role of HPV in cutaneous keratinocyte tumors development has been highlighted [25]. Beta-HPVs have been reported as the main etiologic agent of SCC in patients with *epidermodysplasia verruciformis* (EV) and organ transplant recipients [26]. Several studies have shown that samples from KA contain HPV DNA in more than 90% of cases, unlike well-differentiated SCC, suggesting a co-factor role for HPV [27].

An association between SCCs and beta-HPV infections has been reported [28]. However, their role in NMSC progression is still unclear [28].

In a recent systematic review, 321 KAs were analyzed for the presence of different HPV genotypes, detecting a high presence of beta genus types (50.31%) and an equal proportion between alpha (24.84%) and gamma (24.84%) genera [25]. However, most of the HPV types found in NMSC were the consequence of multiple infections with different HPV types, which were detected at very low viral loads (<1 copy/1000 cells), depending on the polymerase chain reaction (PCR) primers, which raises the issue of bias in detecting only HPV types with high similarity to the primers employed, potentially overlooking others [29].

## 4. Clinical Manifestations

KA is usually a solitary and sporadic tumor [2]. It can vary from a few millimeters up to more than 2 cm in KA *centrifugum marginatum* [3]. KA usually starts as a small papule, while the mature KA is a dome-shaped, well-demarcated umbilicated nodule with a hyperkeratotic plug in the center [2]. KA usually arises on sun-exposed areas and evolves in three clinical stages, namely proliferative, mature, and resolving stage [2]. This process usually occurs within 4 to 6 months and can lead to atrophic hypopigmented scars [2]. Rarely, solitary KA can arise on mucous membranes, mostly in the oral cavity [30,31,32,33,34,35,36,37]. Several studies suggest that KA arises more frequently on non-chronically sun-damaged skin (i.e., chest or arms) while SCC always arises in the context of severely photodamaged skin, usually surrounded by actinic keratosis and/or associated with a field of cancerization. According to these proposed classifications, KA can be clinically recognized as a rapidly growing nodule with a central crateriform area not surrounded by signs of the field of cancerization, while well-differentiated SCC develops upon sun damage and actinic keratosis, proposing a possible different etiopathogenesis between the two tumors.

Multiple KAs are rare and can be sporadic or familial. Multiple KAs *centrifugum marginatum* belong to the first group and are characterized by a coral reef-like appearance [38]. Rarely multiple KAs can be associated with *prurigo nodularis* [39]. Multiple familial KAs can be described in multiple MSSE, an autosomal-dominant skin cancer condition characterized by multiple SCC-like locally invasive cutaneous tumors, also known as Ferguson-Smith disease [40]. Furthermore, patients affected by generalized eruptive KA (GEKA) of Grzybowski develop multiple eruptive KAs, especially on sun-exposed areas, such as the face and the upper trunk, and on intertriginous areas [41,42]. Finally, several KAs develop in patients with multiple familial KAs of Witten and Zak type, a disease that is still not well characterized and combines clinical features of MSSE and GEKA in the same patient [43,44].

## 5. Dermoscopy

**Figure 2:**
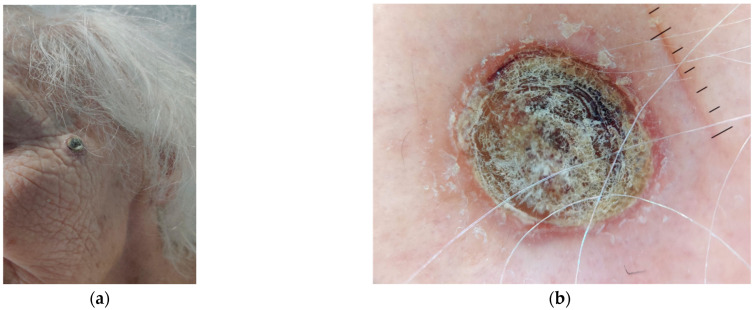
Rapidly growing keratoacanthoma on the left zygomatic-temporal region of an 86-year-old woman. (**a**) Clinical presentation showing a dome-shaped lesion with a central keratin-filled crater. (**b**) Dermoscopic image revealing a central amorphous keratin mass surrounded by a thin erythematous rim.

Dermoscopic examination of KA may present several distinct features that help in differentiating it from other skin tumors (Figure 2) [45]:

1. Central Keratin Plug: One of the hallmark features of KA is the presence of a central, well-defined, yellowish or whitish keratin plug. This central crater is visible in most cases and is a key diagnostic feature.
2. Rolled Border: The lesion typically displays a smooth, well-demarcated, and elevated border that is slightly rolled. This appearance is characteristic of KA and helps in differentiating it from other tumors that may have irregular borders.
3. Vascular Patterns: Dermoscopy often shows vessels that are linear, dotted, or hairpin-shaped in the peripheral areas of the lesion. These vessels may be more prominent around the edge of the keratin plug, contributing to the lesion’s raised appearance. Inflammatory and hyperplastic changes in the epidermis contribute to these vascular patterns.
4. Homogeneous Pink or White Background: The background of the lesion may show a homogeneous pink or white color due to the underlying inflammatory changes and epithelial proliferation. This feature, when seen with the characteristic keratin plug, helps in the diagnosis.
5. Yellowish Structures: Apart from the keratin plug, the lesion may exhibit yellowish areas or streaks within the lesion, which correspond to the presence of keratinized material beneath the surface.
6. Regular Pigment Pattern: In most cases, the dermoscopic pattern of KA is regular, with no irregular pigment network or atypical vessels, which further helps to differentiate it from malignancies such as melanoma.

Dermoscopically, it is not easy to differentiate KA from SCC because they share some common features [46]. Keratin has the highest sensitivity to differentiate KA and SCC from other amelanotic nodules, while white circles have the highest specificity, and they are typically detected in SCC tumors but not in KA. Another important clue for differentiating SCC from KA is the predominantly white color, more frequently detected in SCC, which histologically corresponds to fibrosis and hyperkeratosis, while white circles correspond to the invasion of hair follicles by the tumor. These dermoscopic findings, together with dot vessels, are helpful for the diagnosis of KA and SCC [45].

## 6. Reflectance Confocal Microscopy (RCM) and Line-Field Confocal Optical Coherence Tomography (LC-OCT)

As previously explained, KA is a rapidly growing epithelial neoplasm that often poses diagnostic challenges due to its clinical and histological similarities with well-differentiated SCC. Non-invasive imaging techniques such as reflectance confocal microscopy (RCM) and line-field confocal optical coherence tomography (LC-OCT) have emerged as valuable tools for the real-time, in vivo evaluation of skin tumors, offering near-histologic resolution without the need for tissue excision.

RCM allows a face imaging of the epidermis and superficial dermis, generally reaching a maximum depth of approximately 200–250 µm. This limitation in penetration depth may hinder complete visualization of thick lesions such as KA, which often exhibits marked central hyperkeratosis and deep epidermal proliferation. Nevertheless, RCM can provide useful information, particularly at the lateral and superficial portions of the tumor [47]. On RCM analysis, KA typically presents with epidermal hyperplasia and architectural disarray, often accompanied by a large, highly reflective central structure corresponding to the compact keratin plug. The surrounding keratinocytes may appear atypical, yet they tend to preserve an organized honeycomb pattern, distinguishing KA from more aggressive forms of SCC. Vascular features such as dilated and tortuous capillaries within the dermal papillae, as well as perivascular inflammatory infiltrates, are frequently observed and may support the diagnosis [48].

**Figure 3:**
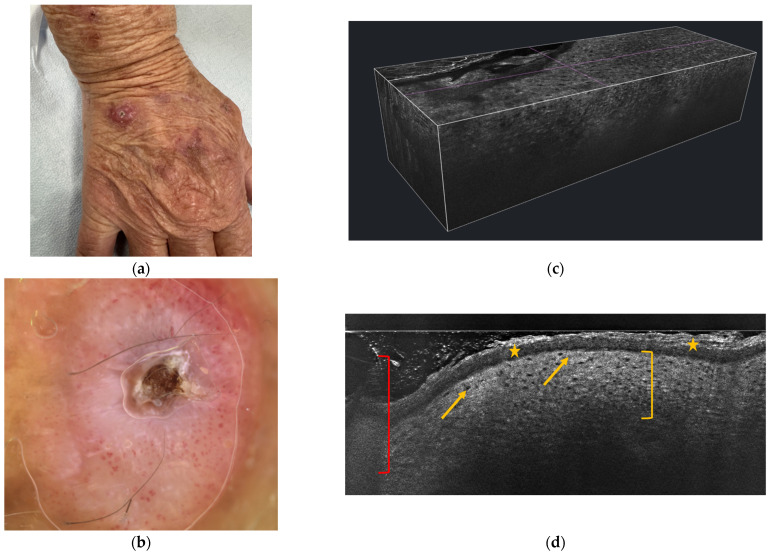
Rapidly growing keratoacanthoma on the dorsum of the right hand in a 78-year-old woman. (**a**) Clinical presentation showing an erythematous nodule with a central keratinous core. (**b**) Dermoscopic image highlighting a central keratin mass, surrounded by white structureless areas and peripheral dotted or hairpin vessels. (**c**) 3D reconstruction of the lesion using line-field optical coherence tomography (LC-OCT). (**d**) Vertical mode LC-OCT image showing marked hyperkeratosis and parakeratosis (yellow stars), atypical keratinocytes (yellow bracket), a central keratinous core (red bracket), and a preserved dermo-epidermal junction (yellow arrows).

On the other hand, LC-OCT, with its capacity to acquire both vertical (B-scan) and horizontal (*en face*) images and a penetration depth of up to 500 µm, offers a more complete structural assessment of KA compared to RCM [49]. The hallmark feature of KA in LC-OCT analysis is the presence of a cup-shaped epidermal invagination filled with a hyperreflective material, corresponding to the crateriform morphology and central keratin seen histologically. The surrounding epidermis typically appears markedly thickened, with abrupt lateral transition zones separating the lesion from the adjacent normal skin. In the vertical plane, broad strands and islands of keratinocytes can often be seen extending downward into the dermis, mimicking the appearance of histologic sections (Figure 3). Although cytological details are not as clearly defined in LC-OCT as in RCM, the architectural pattern is usually well visualized and can aid in distinguishing KA from invasive SCC [50].

Despite their utility, both RCM and LC-OCT have inherent limitations that must be considered. The depth of penetration may be insufficient to evaluate the full extent of the lesion, particularly at the base, and the dense keratin content can attenuate signal penetration, reducing image quality. Furthermore, distinguishing KA from well-differentiated SCC remains challenging, especially in cases where the imaging captures only a portion of the lesion. The interpretation of images is also observer-dependent and may vary based on the examiner’s level of experience and training.

Nonetheless, these techniques can serve as helpful adjuncts in the clinical evaluation of KA, especially in cases where biopsy is contraindicated, where the lesion is in regression, or when monitoring over time is required. The correlation between imaging findings and histopathology, particularly regarding the overall architecture, further supports their integration into the diagnostic pathway for KA.

## 7. Histology of Keratoacanthoma

Differentiating KA from well-differentiated SCC on routine histology can be tricky due to shared features. Indeed, shave or small punch biopsies fail to include the complete tumor architecture [51]. KA histopathological features depend on the development phase, namely, proliferative, mature, and regressive phases [52]. The proliferative phase is characterized by marked hyperkeratosis and proliferation of pale squamous cells in lobules that can resemble distorted infundibular structures, which become more cystic and hyperkeratotic in a later stage, coalescing into a central keratin plug [53]. In the mature stage, most peripheral tumor islands infiltrate the edges of the central mass [53]. Typically, the peripheral keratinocytes show enlarged, pink, glassy-appearing cytoplasm, a low nuclear-to-cytoplasmic ratio, and minimal nuclear atypia [53]. In addition, a mixed infiltrate of inflammatory cells can be commonly detected [53]. Regressing lesions show a well-formed crater of keratin with thinning of the surrounding squamous epithelium, reduced squamous lobules, and underlying dermal fibrosis [53]. The most significant histopathological features that help to confirm the diagnosis of KA are symmetry, the absence of extension beyond the sweat glands, lack of infiltration or desmoplasia, and the little-to-moderate nuclear atypia [54]. Some cases of metastatic KA have been reported in the literature [55]. However, it could be possible that in these cases either a SCC has arisen within a KA [56] or a SCC with a distinct follicular pattern of differentiation was misdiagnosed as KA [57]. In addition, it has been reported that immunocompromised patients with large KA have developed metastasis [58].

## 8. Human Papillomavirus and Carcinogenesis

HPV belongs to the *Papillomaviridae* family, which includes more than 440 genotypes of small DNA viruses, classified into five genera: α, β, γ, μ, and ν [59]. Only a few of them, mainly within α and β genera, can cause significant risks to human health and have received the most attention from researchers [60].

HPV particles are characterized by the presence of circular, 7.9 kb double-stranded DNA included in a nonenveloped icosahedral capsid [61,62]. The HPV genome encodes eight main expressed proteins: the core proteins involved in genome replication (E1 and E2), the core proteins involved in virus assembly (L1 and L2), and the accessory proteins (E4, E5, E6, and E7) [63]. E6 and E7 are considered the major HPV oncoproteins, required to establish persistent infection and propagation of both α and β HPVs and for malignant transformation of the host cell [64,65] and thus represent attractive antitumoral drug targets [66,67,68].

The types α and β of HPV show significant differences in tissue tropism: β types can infect only the skin, whereas α types predominantly infect mucosal tissues. Among α types, it is important to distinguish between high-risk (HR) αHPVs, such as 16, 18, 31, 33, 35, 39, 45, 51, 52, 56, 58, and 59 genotypes, which show a tissue tropism limited to mucosal tissues, and low-risk (LR) αHPVs, which can infect both cutaneous and mucosal tissues. Generally, infections sustained by β types remain subclinical, but certain infections, mainly those caused by HPV5 and HPV8, can become clinically relevant, leading to the development of NMSC in high-risk patients (e.g., immunocompromised organ-transplant individuals or patients affected by the genetic disorder EV) [69,70,71].

**Figure 4:**
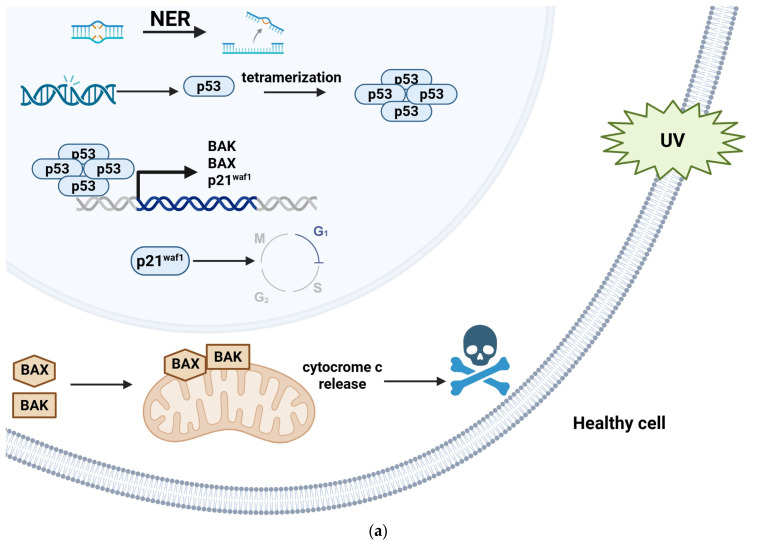

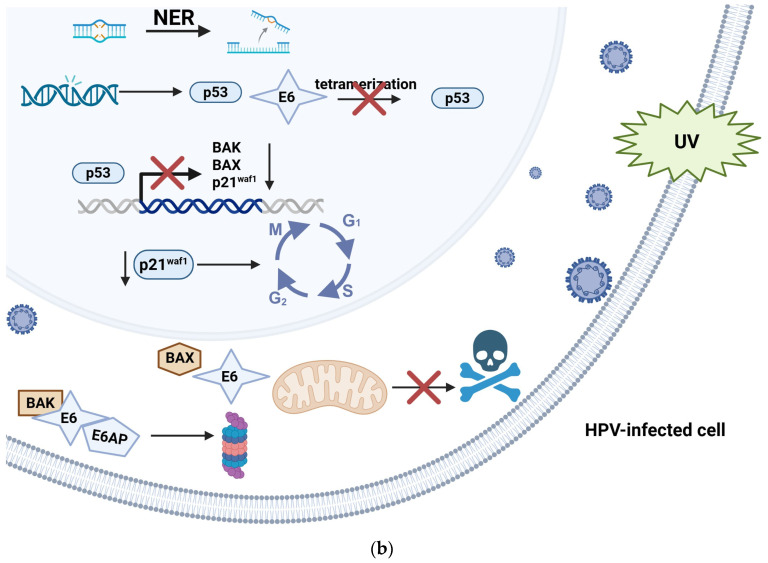
Best-characterized molecular mechanisms involved in carcinogenesis occurring in HPV-infected skin cell. UV radiation induces damage on DNA, which is normally repaired by the Nucleotide Excision Repair (NER) pathway. If NER fails and the DNA damaged is maintained, in the healthy skin cells (**a**) p53 undergoes some post-translational modifications leading to the assembly of a tetramer, resulting in induction of its transcriptional activity, allowing the expression of the cell-cycle inhibitor p21^waf1^, which mediates the cell cycle block in G1 phase, and proapoptotic BAX and BAK proteins. When the NER pathway fails in the skin cell infected by β-HPV (**b**), the presence of E6 interferes with the assembly of p53 monomer into tetramer, finally resulting in loss of apoptotic pathway activation and cell cycle progression. E6 also interferes in a not fully understood way with BAX and, by recruiting E6AP ubiquitin-ligase induces BAX proteasome-mediated degradation.

HR-αHPVs are strongly carcinogenic, whereas βHPVs are not intrinsically carcinogenic but behave as facilitators for cancer initiation [72,73]. As is well-known from the literature, UV-B rays (290–320 nm) are considered strong physical factors damaging macromolecules (i.e., DNA, proteins, lipids) and, in turn, cell integrity [74]. The result of UV-induced DNA damage is represented by specific photoproducts [74], normally removed by the Nucleotide Excision Repair (NER) pathway [75]. If not properly repaired, the photoproducts can lead to mutations [76]. DNA damage response activates the oncosuppressor proteins p53 and p21^waf1^ that arrest the cell cycle. The p53 protein undergoes some post-translational modifications, which regulate the assembly of p53 monomer into a tetramer, inhibit its degradation, and induce its transcriptional activity [77]. P53 has been extensively reported as the best-characterized target of HPV E6 oncoprotein [78]. The HR-αHPV E6 oncoprotein promotes degradation of p53 via the interaction with the E6-associated protein (E6AP) ubiquitin ligase, resulting in the proteasome-mediated degradation of p53 [79,80,81]. In contrast, most of the βHPV E6 oncoproteins inhibit the assembly of the p53 monomer into a tetramer [82,83] (Figure 4). However, some important exceptions have been reported: E6 protein from HPV49, HPV75, and HPV 76 genotypes behave similarly to αHPV E6 protein [84]; E6 protein produced by HPV38 and HPV92 can interact with E6AP and stabilize p53 [85,86]. Moreover, the stabilization of wild-type p53 mediated by E6 from HPV38 results in the accumulation of ΔNp73 [87], which is the carcinogenic isoform of p73 and works as an antagonist of p53 itself [88]. Other intracellular targets for βHPV E6 oncoprotein have been extensively investigated: BAX and BAK are two proapoptotic factors belonging to the BCL-2 family involved in mitochondrial membrane permeabilization occurring during the apoptotic process [89]. βHPV E6-induced, proteasome-mediated degradation of BAK is the result of its interaction with βHPV E6 in an E6AP-dependent manner [90,91,92]. Conversely, the exact mechanism of βHPV E6-induced degradation of BAX has not yet been elucidated.

The other major HPV oncoprotein is E7. For HR-αHPV types, the interactions of E7 with its targets, mainly the retinoblastoma protein (pRb) [93], have been extensively investigated and characterized. In the case of HR-αHPV types, the interaction with pRb triggers its proteasome-mediated degradation and abrogates its negative regulation of cell cycle progression in infected cells. For βHPV genotypes, the effects of E7 interactions are less well understood, but in general do not seem able to interfere with pRb activity in the infected cell, with the only exception of HPV38, whose E7 oncoprotein shows the ability to interact with pRb with an affinity quite similar to that shown by HR-αHPV E7, and to promote its destabilization [94].

Overall, βHPV represents a cofactor that can enhance the effects of UVB damage. Indeed, the presence of βHPV confers resistance to the cell towards apoptosis as well as increases the susceptibility to UVB-mediated DNA damage [95]. The role of βHPV in the pathogenic mechanisms of keratinocyte skin cancers remains elusive but is supported by increasing evidence. A systematic review reported βHPV detection in several skin lesions, including KA [25]. HPV detection rates vary across studies, depending on the sensitivity of employed detection techniques. Forslund et al. [96] detected HPV DNA, by means of PCR, in 51% of KA samples, whereas Baek et al. used next-generation sequencing techniques and detected HPV in 11% of KA samples [97]. Data regarding the proliferative state of these lesions would have been valuable to further clarify the HPV role in KA; however, such information is lacking in the current literature.

## 9. Therapy

KA can regress spontaneously, and a watch-and-wait approach can be a valid option in several cases, especially in elderly patients [98]. However, the first-line therapy for KA remains the complete surgical removal. However, about 8% of KAs may exhibit a recurrence after complete surgical excision [98]. Radiotherapy (RT) and cryotherapy are physical therapies for treating KAs. Although the effectiveness of RT on KA has been demonstrated [99,100,101], RT is rarely used. Indeed, RT is not indicated for younger patients, and the several doses required make it burdensome for older patients. Furthermore, RT may induce eruptive KAs [102]. However, RT could be an option for cosmetically sensitive, non-operable regions. Although cryotherapy has not been widely studied as a therapeutic option, its effectiveness has been reported in about 87% of KAs [103]. Photodynamic therapy (PDT) also belongs to the group of physical therapies. However, only limited data are available on the use of PDT for KAs. On the one hand, PDT has been reported as effective in treating KAs, but on the other hand, the development of KAs after PDT has been reported [104]. The argon lasers have also been used for KAs. Solitary KAs of the face and ears were successfully treated in 65% of the patients, while 35% achieved healing with mild scarring [105]. Topical 5-fluorouracil (5-FU) and imiquimod represent two valuable alternatives for KAs. 5-FU is an inhibitor of thymidylate synthase, disrupts DNA synthesis, and leads to lethal DNA damage [106]. Topical 5-FU has a good safety profile and could be considered as a first-line treatment for KAs, particularly in cosmetically sensitive areas [107]. Indeed, the use of topical 5-FU led to a complete resolution of the lesion within six weeks in a retrospective analysis [107]. 5-FU can also be used for intralesional applications [108]. An average of three injections in three weeks has been reported as effective [108]. Furthermore, 5-FU has been effectively used in combination with systemic retinoids [109] and Er:YAG lasers [110]. Topical 5% imiquimod, a toll-like receptor 7 and 8 agonist able to stimulate immune response, also represents an effective alternative for treating KAs. However, imiquimod should be applied for 9–11 weeks, three to four times a week, to obtain a complete remission [111]. Intralesional administration of MTX is considered another alternative to surgery. MTX is an inhibitor of dihydrofolate reductase, an enzyme which converts dihydrofolic acid to tetrahydrofolic acid, a cofactor in methyl transfer reactions, finally leading to inhibition of thymidylate, purine, and DNA biosynthesis [112]. Nofal et al. observed a complete clearance of the lesion in 70% of treated patients after 5 intralesional administrations of MTX [113]. In addition, Yoo et al. reported a clearance rate of 91% after 2 to 7 injections in a retrospective study on 11 patients [114]. Furthermore, Annest et al. [115] reported in a review that intralesional MTX achieved resolution in 92% of patients, requiring an average of two injections 18 days apart. However, an important limit of this therapeutic option is lack of a standardized protocol.

Systemic therapies for KAs include retinoids and erlotinib. Systemic retinoids act with several mechanisms, including inhibition of keratinization and Wnt-related KAs proliferation, modulation of terminal differentiation of epidermal cells, and an increase in both IL-2 production and mitogen-induced lymphocyte proliferation [5]. Oral retinoids represent a valuable option for generalized eruptive KAs of Grzybowski and Ferguson-Smith disease [116,117,118]. Erlotinib, an epidermal growth factor receptor (EGFR) inhibitor, has also been proposed for the treatment of resistant, multiple KAs [119]. However, experience with it is still limited. A summary of the above-described therapeutic options for the treatment of KAs is provided in Table 1.

Surgical excision remains the gold standard treatment for KA, with both definitive histopathological diagnosis and good therapeutic outcomes. Recurrence is not common if margins are adequate, even though scarring and wound complications may occur, particularly in cosmetically sensitive areas. Mohs micrographic surgery has similar effectiveness and better tissue preservation and cosmetic outcomes. Nevertheless, it is more expensive, and its availability is still limited [98].

Although RT results are effective, it is only rarely used, because of costs, requirement for multiple sessions, and risk of long-term cutaneous damage. For this reason, it is generally reserved for patients unfit for surgery [100].

Non-surgical therapeutic approaches have been described and they show variable success. The topical administration of 5-FU or imiquimod can induce regression in selected cases, even though evidence is still limited and local inflammatory reactions often occur [107,115]. Intralesional administration of drugs, mainly MTX, have shown promising effectiveness, are relatively cheap, and can be useful for elderly patients with comorbidities or for lesions in cosmetically sensitive areas. Nevertheless, they require repeated injections and carry a small risk of systemic toxicity [108,113].

Although KA may spontaneously regress, a strategy of watchful waiting is rarely appropriate given the clinical and histopathological overlap with invasive SCC and the potential for undertreatment. Overall, excision—conventional or Mohs—remains the most reliable option, while less invasive modalities may be considered in selected patients where surgical risks, cosmetic considerations, or comorbidities outweigh the benefits of standard treatment.

## 10. Anti-HPV Vaccines

Three main vaccines are commercially available and approved to prevent cervical cancer caused by the most common HR-HPV genotypes: Gardasil^®^, Gardasil^®^9, and Cervarix^®^. They all are based on non-infectious virus-like particles (VLPs) containing the major capsid protein L1 from the specific HPV genotype they target and are approved for the prevention of premalignant ano-genital lesions (cervical, vulvar, vaginal and anal), cervical and anal cancers causally related to certain oncogenic HPV types [120].

**Figure 5:**
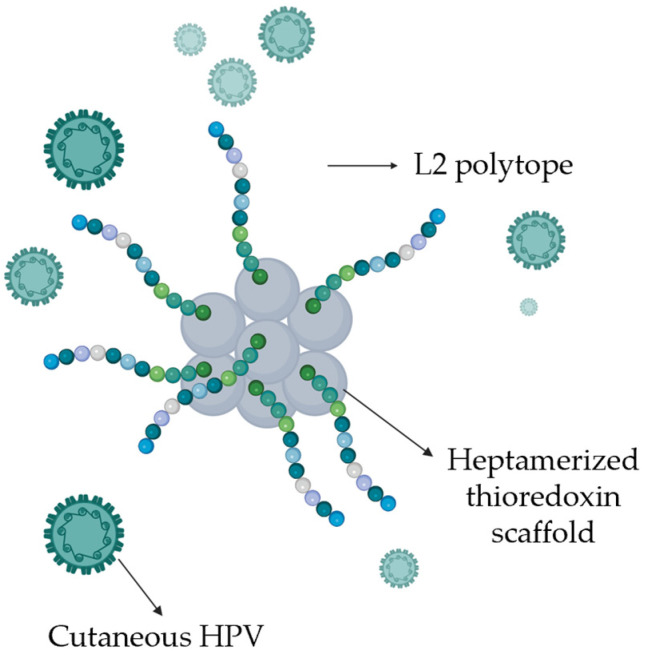
Schematic representation of new promising vaccine candidates, containing the L2 polytope of the cutaneous HPV types, fused to the thioredoxin scaffold.

The employment of the three available vaccines, which are both safe and effective, had a great success for the prevention of HPV-related cancers. Nevertheless, being based on L1, which is poorly conserved among different HPV types, they can target just the specific corresponding genotype, with incomplete cross-protection [121]. Therefore, many efforts have been made by researchers to develop novel strategies to achieve a broader vaccine efficacy across HPV genotypes. In this regard, a research group developed other promising vaccine candidates, containing the L2 polytope, i.e., a synthetic antigen composed of concatenated conserved epitopes from the L2 protein of the cutaneous HPV types, fused to the thioredoxin scaffold (Figure 5). In contrast to L1, L2 is highly conserved among both mucosal and cutaneous HPV genotypes. These candidates were tested in vivo in both mice and guinea pigs, resulting in a broad immunization against 19 cutaneous HPV genotypes. Interestingly, the investigated vaccine prototypes elicited cross-protective neutralizing antibodies against multiple mucosal HPV types, including the HR HPV16 and HPV18, as well as some LR genotypes [122]. However, it is mandatory to underline that, since KA is generally considered a self-limiting skin lesion with negligible tumor-associated mortality, the rationale behind the efforts to develop new vaccines primarily lies in the prevention of HR-αHPV genotypes infections, which are responsible for several cancers with a significant tumor-associated mortality. Nevertheless, the development of broader vaccines based on the highly conserved L2 epitopes might also provide further advantages by achieving cross-protection against cutaneous HPV types. This goal should be particularly desirable mainly for immunocompromised high-risk patients.

## 11. Materials and Methods

Given the narrative nature of the present review, no predefined inclusion or exclusion criteria were established prior to the literature search.

A comprehensive search of the PubMed and Scopus databases was conducted to identify relevant publications. The key terms used for the search strategy included: “keratoacanthoma” OR “KA” AND “pathogenesis” OR “etiology” OR “human papillomavirus” OR “HPV” OR “management” OR “treatment” OR “therapy” OR “diagnosis” OR “dermoscopy” OR “reflectance confocal microscopy” OR “RCM” OR “line-field confocal optical coherence tomography” OR “LC-OCT” OR “Histopathology”. No language or publication date restrictions were applied. The database search was performed from inception to 7th August 2025. Additional relevant articles were identified by manual cross-referencing from the bibliographies of retrieved papers. Preference was given to peer-reviewed original articles, systematic reviews, meta-analyses, and significant case series that addressed the epidemiology, clinical presentation, diagnostic tools, histopathological features, molecular mechanisms, and therapeutic strategies for KA.

The following PICO (Population, Intervention or exposure, Comparison, Outcome) algorithm was applied to guide the literature selection: (i) Population: patients with KA (solitary, multiple, or eruptive forms); (ii) Intervention/exposure: diagnostic and therapeutic approaches, as well as studies on etiopathogenesis and HPV involvement; (iii) Comparator: other cutaneous epithelial tumors, particularly well-differentiated SCC; (iv) Outcome: advances in understanding pathogenesis, improvements in diagnostic accuracy, and evaluation of therapeutic efficacy.

## 12. Conclusions

KA is a rapidly growing epithelial neoplasm that often poses diagnostic challenges due to its clinical and histological resemblance to well-differentiated SCC. Accurate diagnosis benefits from various non-invasive imaging techniques, such as dermoscopy, RCM, and LC-OCT, which allow for near-histologic, real-time in vivo evaluation, although they are limited by penetration depth and keratin density. Histopathological examination remains essential, with key features including symmetry, absence of extension beyond sweat glands, lack of stromal infiltration, and minimal nuclear atypia helping to distinguish KA from SCC. However, inaccurate biopsies may lead to misdiagnosis and overtreatment. HPVs, particularly cutaneous β types, act as cofactors in skin carcinogenesis by interfering with cellular responses to UV-induced DNA damage and promoting lesion development. The complex interplay between viral factors, UV exposure, and host susceptibility continues to be an important area of research. Surgical excision remains the first-line treatment for KA, though non-surgical options—including radiotherapy, cryotherapy, photodynamic therapy, topical agents such as 5-fluorouracil and imiquimod, and systemic therapies like retinoids and erlotinib—offer valuable alternatives in select cases, especially for multiple or difficult-to-treat lesions. Available prophylactic HPV vaccines, targeting HR mucosal genotypes, have substantially advanced cancer prevention, while novel vaccine candidates based on the conserved L2 protein hold promise for broader protection against both cutaneous and mucosal HPV types.

Integrating advances in diagnostic approaches, understanding of pathogenetic mechanism, and therapeutic strategies is critical for optimizing the management of KA and HPV-associated lesions, to improve diagnostic accuracy, minimizing overtreatment, and developing more effective prevention measures.

Some important challenges still remain, mainly the distinction between KA and well-differentiated SCC. In the future this aim should be achieved by integrating advanced imaging techniques and histopathology. More in depth research on β-HPV genotypes should clarify some incompletely understood carcinogenesis mechanisms and open the road to identifying new biomarkers. Finally, beyond surgery, the validation of less invasive therapeutic approaches and effective prevention strategies for high-risk patients could broaden management options for this challenging tumor.

## Figures and Tables

**Table 1:** Summary of currently available therapeutic options for the treatment of KA.

Therapy Type	Description		Mechanism		Effectiveness
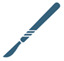 surgical	Complete surgical removal		Surgical removal with histopathological control		Gold-standard, but ~8% recurrence
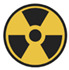 physical	Radiotherapy		Targeted destruction of lesions via	Energy	Effective, but rarely used
	Cryotherapy			Cold	Effective (~87%), but not widely studied
	PDT ^1^			Locally activated drug	Limited available data
	Argon laser			Laser	Effective for solitary KA ^2^
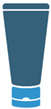 topical	Local application of	5-FU ^3^	Inhibition of DNA synthesis		Very effective, first-line treatment
		Imiquimod	Local immune response activation		Effective
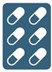 systemic	Retinoids		Control of proliferation, differentiation, and immune response		Valuable option for generalized eruptive KA ^2^
	Erlotinib		EGFR ^4^ inhibition		Still limited experience
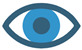 active surveillance	Watch-and-wait approach		KA can regress spontaneously		Rarely appropriate, but can be considered as a strategy mainly for elderly patients

^1^ PDT: Photodynamic therapy. ^2^ KA: Keratoacanthoma. ^3^ 5-FU: 5-fluorouracil. ^4^ EGFR: Epidermal Growth Factor Receptor.

## Data Availability

No new data were created or analyzed in this study. Data sharing is not applicable to this article.

## Abbreviations

The following abbreviations are used in this manuscript:

KA	Keratoacanthoma
SCC	Squamous Cell Carcinoma
MTX	Methotrexate
RCM	Reflectance Confocal Microscopy
LC-OCT	Line-Field Confocal Optical Coherence Tomography
HPV	Human Papillomavirus
UV	Ultraviolet
MSSE	Multiple Self-healing Squamous Epithelioma
EV	*Epidermodysplasia Verruciformis*
GEKA	Generalized Eruptive Keratoacanthoma
HR	High-Risk
LR	Low-Risk
NER	Nucleotide Excision Repair
E6AP	E6-Associated Protein
pRb	Retinoblastoma protein
RT	Radiotherapy
PDT	Photodynamic therapy
5-FU	5-Fluorouracil
EGFR	Epidermal Growth Factor Receptor
